# Abscopal effect when combining oncolytic adenovirus and checkpoint inhibitor in a humanized NOG mouse model of melanoma

**DOI:** 10.1002/jmv.25501

**Published:** 2019-06-24

**Authors:** Lukasz Kuryk, Anne‐Sophie W. Møller, Magnus Jaderberg

**Affiliations:** ^1^ Targovax Oy, Clinical Science Helsinki Finland; ^2^ Department of Virology National Institute of Public Health – National Institute of Hygiene Warsaw Poland; ^3^ Targovax ASA, Clinical Science Oslo Norway

**Keywords:** adenovirus, apoptosis/cell death, cellular effect, disease control, genetics, immune responses, Immunomodulation, recombinant virus, vaccines/vaccine strains, virus classification

## Abstract

Melanoma, an immunogenic tumor, is the first indication where oncolytic viruses are now becoming part of clinical practice. ONCOS‐102, a transgened adenovirus, has shown to act as a primer of relevant tumor targeting immune cells both in preclinical and clinical melanoma studies. Strategies to augment its effectiveness warrant investigation. Combination therapy of ONCOS‐102 with the checkpoint inhibitor (CPI) pembrolizumab was evaluated in a quasi‐human animal model, the humanized NOG mouse model. A dosing schedule of the combination, beginning the CPI concurrently with the oncolytic viral therapy and continuing the CPI treatment, appeared to induce an abscopal effect in untreated tumor lesions. Concurrent combination therapy with checkpoint inhibitors may improve the induction of antitumor immune responses of ONCOS‐102.

## Highlight

- The combinatory therapy of ONCOS‐102 and CPI, appeared to induce an abscopal effect in untreated tumor lesions.
- The data support the development of ONCOS‐102 with checkpoint inhibitors for the treatment of malignant cancer diseases.

## 1 INTRODUCTION

Patients with metastatic melanoma (MM) with local or distant tumor nodules have a 5‐year survival rate of 63% and 16% to 20%, respectively.^1^ Oncolytic adenoviruses execute their antitumor activities by multiple mechanisms that harness the interactions between virus, the immune system, and dysfunctional cell cycle and protein expression in cancer cells.^2^ Advantages of oncolytic viruses include selective replication in tumor cells; lysis of tumor cells causing an immunogenic cell death; viral spread that can augment the initial potency of the inoculum; excellent safety profile with minimal effect on surrounding and distant healthy cells and on healthy, rapidly growing cells (eg gastrointestinal tract, hematopoietic system, adult stem cells); and genomic capacity to deliver exogenous proteins.^2^, ^3^, ^4^, ^5^, ^6^, ^7^, ^8^, ^9^ Local secretion of granulocyte macrophage colony stimulating factor (GM‐CSF) by oncolytic viruses can increase recruitment, activation, and differentiation of monocytes/macrophages, and can augment local antitumor immune responses.^2^, ^10^, ^11^ Although the efficacy of oncolytic viruses given monotherapeutically have been proven,^12^ additional treatment strategies are needed to develop further improved response rates.^2^ Since oncolytic adenoviral treatment induced programmed death‐ligand 1 (PD‐L1) expression in mesothelioma tumors in a clinical trial,^13^ we and others^14^ are investigating the combination therapy of oncolytic adenoviruses and checkpoint inhibitors.

Checkpoint inhibitors targeting programmed cell death protein (PD‐1) on T cells and other immune cells impede the interaction of PD‐1 with their ligands on tumor cells, thereby reactivating the antitumor immune response.^15^, ^16^ Checkpoint inhibitor therapy (eg. pembrolizumab, nivolumab) of solid tumors has greatly extended the survival of 16% to 20% patients with MM^17^ and other solid tumors. To potentially broaden the responsive patient population, we have chosen to assess the combination therapy of the oncolytic adenovirus ONCOS‐102 and the FDA‐approved checkpoint inhibitor, pembrolizumab.

Oncolytic viruses (OVs) have the ability to selectively replicate and lyse cancer cells, spreading within the tumor mass and not significantly harming normal cells. OVs can exhibit natural tumor‐selective tropism or can be genetically modified for cancer cell‐restricted replication.^18^, ^19^, ^20^, ^21^ The oncolytic adenovirus ONCOS‐102 has three modifications that can augment its efficacy against melanomas.^22^, ^23^ Its chimeric adenoviral 3/5 knob targets the frequently overexpressed membrane protein desmoglein on melanoma cells. Its 24 bp deletion in the *E1A* gene limits its replication to tumor cells with an altered Rb pathway.^24^ Its expression of GM‐CSF augments the immunostimulatory microenvironment in the infected tumor. To more closely mimic the induction of human antitumor immune responses, the human hematopoietic stem cell‐engrafted NOG mice (hu‐NOG mice)^25^ were used. Here we modified the dosing schedule to potentially augment the antitumor immune response not only in the treated tumor nodule but also the untreated tumor nodule, with the goal of inducing abscopal effects that are essential for the treatment of MM.

## 2 MATERIALS AND METHODS

### 2.1 Animals

Animal experiments were compliant with EU Directive (63/2010), adhered to the guidelines from Federation of the European Laboratory Science Association (FELASA), were reviewed and approved by the local ethics committee (01_TransCurebioServices‐AB‐01).

NOG mice (age, sex, source) were humanized by intravenous injection of 60 000 cord blood human hematopoietic CD34^+^ stem cells. After 14 weeks, the hu‐NOG mice (average % humanization rate (*H* rate) was 54, Figure S1B) were engrafted with A2058 tumor cells (2 × 10^6^ cells per flank), as described.^26^

### 2.2 Treatment

**Figure 1:**
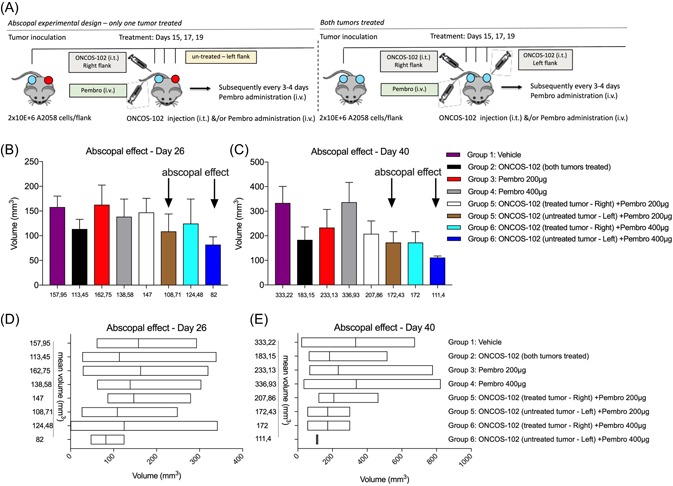
A, Diagram of study design. Effect of combination therapy of ONCOS‐102 and pembrolizumab assessed in a hu‐NOG mouse model of melanoma. B, Tumor volume on day 26, results represents mean ± SEM. C, Tumor volume on day 40, results represents mean ± SEM. D, Tumor volume on day 26, floating bars (min to max), with line at mean. E, Tumor volume on day 40, floating bars (min to max), with line at mean. The differences between MTVs among groups were not statistically significant (NS). i.t., intratumoral; i.v., intravenous; MTV, mean tumor volume; Pembro, pembrolizumab; SEM, standard error of the mean

ONCOS‐102 was grown in A549 cells, harvested, purified as described.^19^, ^22^ The hu‐NOG mice were grouped (n = 6‐8 mice, 12‐16 tumors) for a similar average humanization rate (54%) and mean tumor volume (MTV) of approximately 25 mm^3^. On days 15, 17, and 19, the tumors on the right/left or both sides were injected with 50 µL ONCOS‐102 (2.5 × 10^6^ VP per tumor) or vehicle (sterile PBS). The mice also received intravenous administration of vehicle or pembrolizumab (200 or 400 µg) on days 15, 17, 19 and every 3 to 4 days throughout the study (Table 1, Figure 1A).

**Table 1:** Treatment dosage and schedule

	Treatment modality schedule			
	ONCOS‐102 (2.5 × 10^6^ VP/tumor)		Pembro (i.v.)	Treatment regime
Treatment groups	Left tumor	Right tumor		
1. Vehicle), n = 8 mice/16 tumors	(PBS)	(PBS)	(PBS)	Days 15, 17, 19 intratumoral i.v. on days 15, 17, 19 and every 3‐4 d throughout the study
2. (ONCOS‐102), n = 8 mice/16 tumors	Yes	Yes	No	Days 15, 17, 19 intratumoral
3. (Pembro 200 µg), n = 8 mice/16 tumors	No	No	200 µg	i.v. on days 15, 17, 19 and every 3‐4 d throughout the study
4. (Pembro 400 µg), n = 8 mice/16 tumors	No	No	400 µg	i.v. on days 15, 17, 19 and every 3‐4 d throughout the study
5. (ONCOS‐102 + Pembro 200 µg), n = 6 mice/12 tumors	No	Yes	200 µg	OV: Days 15, 17, 19 intratumoral
				Pembrolizumab: i.v. on days 15, 17, 19 and every 3‐4 d throughout the study
6. (ONCOS‐102 + Pembro 400 µg), n = 6 mice/12 tumors	No	Yes	400 µg	OV: Days 15, 17, 19 intratumoral
				Pembrolizumab: i.v. on days 15, 17, 19 and every 3‐4 d throughout the study

Abbreviations: i.v., intravenous; OV, ONCOS‐102; PBS, phosphate buffered saline.

### 2.3 Read‐out

Tumor dimensions were measured 3× weekly and the volumes calculated as (length × width^2^/2), as described. Body weight loss also was measured 3× weekly and the percent loss compared to baseline calculated accordingly (Figure S1A).

### 2.4 Statistics

Statistical significance was analyzed by using RM‐one‐way analysis of variance followed by a Tukey posttest or T test (Mann‐Whitney test). All statistical analysis, calculations, and tests were performed using GraphPad Prism 8 (GraphPad Software, San Diego, CA). Results are presented as mean ± SEM. All *P* values were two‐sided, considered statistically significant when  ≤0.05.

## 3 RESULTS

We have previously shown that ONCOS‐102 treatment followed by three doses of pembrolizumab can slow or halt the growth of the A2058, A375, and SK‐MEL‐2 melanoma tumor nodules in humanized mice. This schedule of the combination therapy induced a reduction in the untreated left tumor volumes in the SK‐MEL‐2 tumor‐bearing hu‐NOG mouse group but not in the A375 tumor‐bearing hu‐NOG mouse group (unpublished data).

To potentially induce an abscopal effect against A2058‐tumor‐bearing hu‐NOG mice, the dosing schedule of the combination therapy of ONCOS‐102 and pembrolizumab was revised so that both agents were administered on days 15, 17, and 19 with continuation of pembrolizumab administration every 3 to 4 days thereafter (Figure 1 and Table 1).

On day 26, the MTVs (Figure 1B and 1D) of the group 2 of both tumors intratumorally treated with ONCOS‐102 was 11 345 mm^3^ and was lower than mice treated intravenously with pembrolizumab (either 200 or 400 µg, groups 3 and 4), respectively, 16 275 and 13 858 mm^3^ (Figure 1B and 1D). The MTV of the PBS‐injected tumors of the vehicle were 15 795 mm^3^ (group 1).

Tumors on the mice with the right tumor treated with the virus intratumorally plus pembrolizumab intravenously (either 200 or 400 µg, groups 5 and 6) were modestly greater (respectively 147 and 12 448 mm^3^) than the MTV of the ONCOS‐102 treated mice (11 3 45 mm^3^, group 2). Instead, the untreated tumors (left flank) on the hu‐NOG mice with the right tumors treated with ONCOS‐102 followed by pembrolizumab (either 200 or 400 µg, groups 5 and 6) were smaller (respectively, 10 871 and 82 mm^3^) than the MTV of the mice treated with the right tumors in the same group (respectively, 147 and 12 448 mm^3^) and MTV of the ONCOS‐102 treated groups (11 345 mm^3^, group 2). The difference between the MTVs among the groups were not statistically significant.

On day 40, the MTV of the right treated tumors (groups 5 and 6) were larger (respectively, 20 786 and 172 mm^3^) than tumor volumes of the untreated left tumors on the same hu‐NOG mice (respectively, for groups 5 and 6: 17 243 and 1114 mm^3^). Untreated tumors from these groups were also smaller than the MTVs of the mice treated with ONCOS‐102 into two tumors (18 315 mm^3^, group 2). The treatment with pembrolizumab only (either 200 or 400 µg, groups 3 and 4) was less effective than virus alone or combinatory therapies (ONCOS‐102 and pembrolizumab) (Figure 1C and 1E). The differences between MTVs among groups were not statistically significant.

## 4 DISCUSSION

A major goal of immunotherapy is the induction of an abscopal effect so that all tumor lesions including distant metastases are reduced or eliminated. We have shown that three intratumoral treatments with the oncolytic adenovirus ONCOS‐102 followed by three systemic doses of pembrolizumab can slow or halt the growth of the A2058,^26^ A375, and SK‐MEL‐2 melanoma tumor nodules in humanized mice (unpublished data). This schedule of the combination therapy induced a reduction in the untreated left tumor volumes in the SK‐MEL‐2 tumor‐bearing hu‐NOG mouse group but not in the A375 tumor‐bearing hu‐NOG mouse group (data unpublished). Here, we showed that a modified dosing schedule of the combination, beginning the CPI concurrently with the oncolytic viral therapy and continuing the CPI treatment, appeared to induce an abscopal effect in the untreated left tumor nodules of the same size in a humanized A2058 melanoma model.

Several animal studies using different combination therapies and treatment regiments have studied the abscopal effect. A study of combination therapy comprised of oncolytic vaccinia Western Reserve strain and anti‐PD1, anti‐CTLA‐4, or Oxaliplatin showed an abscopal effect in untreated MCA205 Ifnar^−/−^ tumors.^27^ Interestingly, the timing of a single administration of the CPI at 7 days post oncolytic virus treatment significantly increased the efficacy of the combination therapy against the MCA205wt tumor cells in vivo.^27^ The probability and effect size of the abscopal effect was maximized by using the tumor variant which was most susceptible to the oncolytic WR virus (MCA205Ifnar^−/−^), delayed tumor inoculum for the contralateral untreated tumor nodules, and oncolytic virus treatment before administration of checkpoint inhibitor. They showed superior responses when combination therapy in which the anti‐PD1, anti‐CTLA‐4, or the oxaliplatin was administered after rather than before the oncolytic virus.^27^

A phase 1b human trial evaluated the safety of the GM‐CSF‐expressing herpes‐based oncolytic virus tamimogene laherparepvec (weeks 1, 4, 6, every 2 weeks) combined with systemic pembrolizumab (week 6, every 2 weeks) for treatment of MM. This therapy had a similar safety profile to treatment with the individual agents. It also induced a 50% reduction in 82% of injected lesions, 43% of noninjected nonvisceral lesions, and 33% of noninjected lesions.^28^ Interestingly, the antitumor responses did not correlate with baseline CD8^+^ infiltration, PD‐L1 status, nor interferon signature.^28^ As determined by IHC of planned biopsies, the oncolytic herpes virus increased the infiltration of CD8^+^ T cells in patients who responded to the combined therapy.^28^

Combination therapy comprising the oncolytic New Castle Disease Virus and a concurrently administered murine‐specific checkpoint inhibitor‐induced abscopal effect on the untreated tumor in a mouse model of bladder cancer.^29^ On days 7, 9, 11, and 13 after MD49 bladder tumor cells were injected into the flanks of C57BL/6J mice, the right flank tumors were treated with NDV (10^7^ pfu) and the animals concurrently received systemically (intraperitoneally) the anti‐PD‐L1 or anti‐CTLA‐4 antibodies. The MTVs of the untreated tumors were reduced, indicative of an abscopal effect.^29^ These studies show that information from animal studies may be predictive of responses in patients.

Our in vivo study has several limitations. Since each individual NOG mouse is engrafted with mesenchymal and hematopoietic stem cells from a single human umbilical cord blood donor not shared with other mice, the multiple donors for the hu‐NOG mice are likely to have provided a more heterogeneous immune response than inbred mouse strains (allogenic humanization). We expect that the HLA haplotypes of at least some donors were distinct from the A2058 melanoma tumor line. More complete human immune system with human leukocyte antigen‐restricted T cells could be acquired and tested by autologous patient‐specific transplant of peripheral blood mononuclear cells and tumor cells and so provided HLA‐matched tumor cells. This would likely generate a closer representative of patients’ antitumor response. However, the strength of our study using allogenic humanization is the ability to generate multiple hematopoietic lineages including T and B cells, dendritic cells and NK cells. This in turn means comparable tumor inoculums, comparable duration of tumor engraftment for both the treated and the untreated tumors, and the use of a wild‐type human melanoma cell line rather than a cell line with optimized sensitivity to oncolysis.

In conclusion, the data from this study further support the development of ONCOS‐102 and combination therapy of ONCOS‐102 with checkpoint inhibitors such as pembrolizumab for the treatment of malignant cancer diseases.

## CONFLICT OF INTERESTS

The authors declare that there are no conflict of interests.

## Supporting information

Supporting information

Supporting information
