# Supplementary material for: Abscopal effect when combining oncolytic adenovirus and checkpoint inhibitor in a humanized NOG mouse model of melanoma
Source: J Med Virol. 2019 Jun 24;91(9):1702–6. doi: 10.1002/jmv.25501 (PMC6771875; doi:10.1002/jmv.25501)
Supplement: Supplementary file 2 — Supporting information [file JMV-91-1702-s002.docx]

**Supplementary Figure 1**. **A** Percent change in body weight throughout the study. **B** Mice randomization after tumor cell engraftment. Humanization rate (H index) was defined as the ratio of circulating hCD45+/total CD45+ (mCD45+hCD45). Results represents mean +/- SEM.
